# Evaluation of Polyhedral Oligomeric Silsesquioxane Porphyrin Derivatives on Photodynamic Therapy

**DOI:** 10.3390/molecules25214965

**Published:** 2020-10-27

**Authors:** Paolo Siano, Alexis Johnston, Paula Loman-Cortes, Zaneta Zhin, Juan L. Vivero-Escoto

**Affiliations:** 1 Department of Chemistry, University of North Carolina Charlotte, Charlotte, NC 28223, USA; psiano@uncc.edu (P.S.); ajohn391@uncc.edu (A.J.); plomanco@uncc.edu (P.L.-C.); zzhin@uncc.edu (Z.Z.); 2 Nanoscale Science Program, University of North Carolina Charlotte, Charlotte, NC 28223, USA; 3 Center for Biomedical Engineering and Science, University of North Carolina Charlotte, Charlotte, NC 28223, USA

**Keywords:** polyhedral oligomeric polysilsesquioxane, nanoparticles, photodynamic therapy, cancer, porphyrins

## Abstract

Polyhedral oligomeric silsesquioxane (POSS) is a promising scaffold to be used as delivery system. POSS can modify the properties of photosensitizers to enhance their efficacy toward photodynamic therapy (PDT). In this work, we designed, synthesized and characterized five different POSS porphyrin (**POSSPs 1–5**) derivatives containing hydrophobic (**1**–**3**) and hydrophilic (**4** and **5**) functional groups. In general, all the POSSPs showed a better singlet oxygen quantum yield than the parent porphyrins due to the steric hindrance from the POSS unique structure. **POSSPs 1** and **3** containing isobutyl groups showed better PDT performance in cancer cells at lower concentrations than **POSSPs 4** and **5**. However; at higher concentrations, the **POSSP** **4** containing hydrophilic groups has an enhanced PDT efficiency as compared with the parent porphyrin. We envision that the chemical tunability of POSSs can be used as a promising option to improve the delivery and performance of photosensitizers.

## 1. Introduction

Photodynamic therapy (PDT) is a non-invasive technique for the treatment of various cancers with high selectivity, minimal long-term effect and excellent cosmetic appeal [1,2]. The basic working principle for PDT relies on light, oxygen and a photosensitizer (PS), which together are used to generate reactive oxygen species (ROS) that induces cell apoptosis/necrosis. PSs used in PDT (e.g., porphyrins) aggregate in aqueous media due to their poor solubility resulting in self-quenching and decreasing its bioavailability towards cancer cells. PS molecules are chemically modified to change their physicochemical properties to overcome those issues [3]. Nevertheless, the synthetic approaches are usually time-consuming and costly. Therefore, different approaches such as the physical or chemical incorporation of PSs in nanoparticles and polymeric micelles are being explored [4,5,6,7,8].

Polyhedral oligomeric silsesquioxane (POSS) is a class of nanostructured three-dimensional building blocks used to synthesize hybrid materials with a wide variety of biomedical applications such as tissue engineering, dentistry, drug delivery and bioimaging [9,10,11,12,13,14,15,16,17,18,19]. We hypothesize that polyhedral oligomeric silsesquioxane (POSS) compounds can be an attractive alternative to develop customizable scaffolds to functionalize PSs with a broad variety of groups. POSS compounds combined with porphyrins or chlorins have recently been used as scaffolds to develop nanoparticles for photodynamic therapy (PDT). Wu et al. modified POSS with chlorin e6 (Ce6) and polyethylene glycol (PEG) to produce POSS-Ce6-PEG with a high Ce6 content (19.8 wt%) and remarkable anticancer efficiency under light irradiation against HeLa cells and a U14 xenograft mice model [20]. Kim et al. also fabricated nanoparticles using POSS-Ce6 modified with a cancer-targeting peptide. This POSS-Ce6 platform was successfully evaluated in vitro and in vivo against triple-negative breast cancer (MDA-MB-231 cells) [21]. In another approach, Zhang et al. prepared a block copolymer using POSS and 4-vinylbenzyl-terminated tetraphenylporphyrin as the monomers. The self-assembly of this copolymer afforded nanoparticles with high photochemical yield and phototoxicity against a lung cancer model [17]. In a recent report, Wang et al. demonstrated the use of a tetra-PEG-POSS substituted with porphyrin for PDT treatment of cervical cancer in vitro and in vivo. The platform showed higher water solubility, good PDT efficiency and better anticancer performance compared to Foscan [18]. Despite these inspiring works, to the best our knowledge, the literature lacks a systematic investigation that focuses on the effects of different functional groups on the silsesquioxane cage of the POSS-photosensitizer system for PDT.

**Figure 1:**
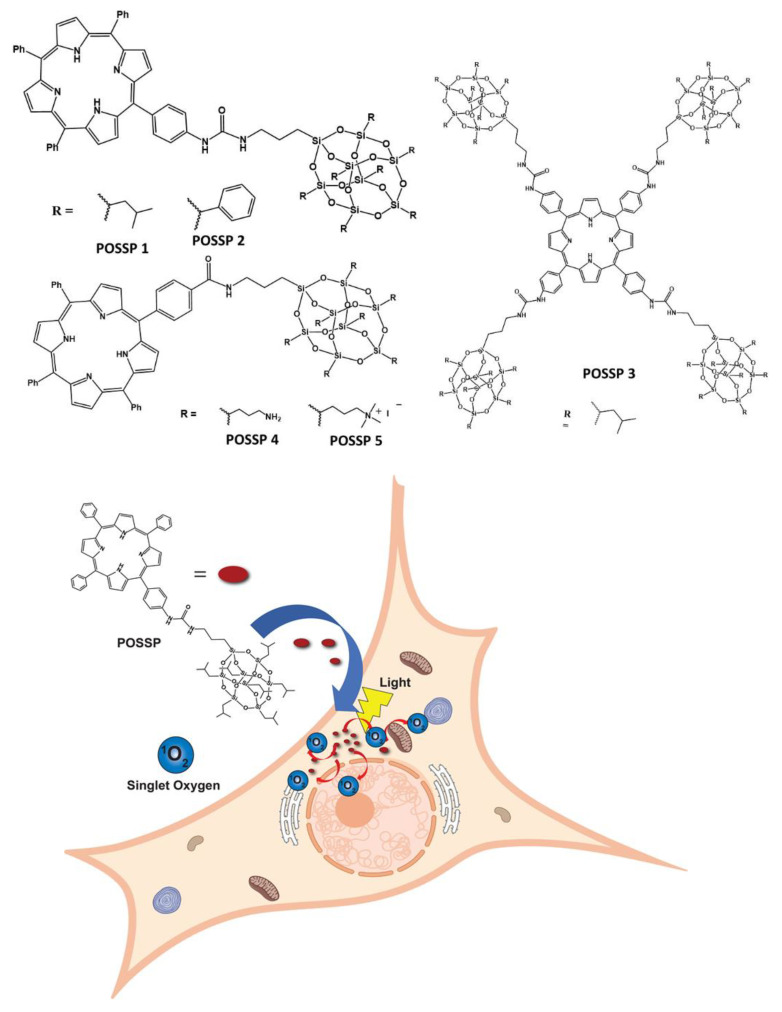
POSSP derivatives synthesized in this work. **POSSPs 1**–**3** contain hydrophobic groups. **POSSPs 4** and **5** are functionalized with hydrophilic moieties. Schematic representation of the internalization of POSSPs in cancer cells and PDT effect.

Herein, we report on the performance of five polyhedral oligomeric silsesquioxane porphyrin (POSSP) compounds toward PDT (Figure 1). These POSSP molecules were rationally designed to contain different functional groups such as alkyl (isobutyl, **POSSP 1**), aromatic (phenyl, **POSSP 2**), amino (**POSSP 4**) and trimethyl-ammonium (**POSSP 5**), which can have different interactions with cancer cells due to their hydrophobic/hydrophilic properties. Moreover, to evaluate the steric effect, a tetra-POSS substituted compound was also designed (**POSSP 3**). These POSSPs were synthesized in a multi-step synthetic approach and characterized using multiple spectroscopic techniques. The photophysical and photochemical features of the POSSPs such as fluorescence and singlet oxygen quantum yields were also determined. The singlet oxygen quantum yield values for POSSP derivatives were higher in comparison with the parent porphyrins. This is most likely due to the steric hindrance effect associated with the unique structure of POSS. The PDT properties of these molecules were evaluated in a triple-negative breast cancer cell line, MDA-MB-231. The PDT performance of these POSSPs at lower concentrations was functional group-dependent, with **POSSPs 1** and **3** containing hydrophobic groups showing a higher phototoxicity. We associated this PDT efficacy with better photochemical properties. Nevertheless, when the PDT performance is carried out at much higher concentrations, these POSSPs cannot be further solubilized, but **POSSPs 4** and **5** containing hydrophilic groups showed a good PDT outcome. In summary, these results demonstrate that the photophysical, photochemical and in vitro PDT properties of POSSPs can be modified based on different functional groups in the POSS molecule.

## 2. Results and Discussion

### 2.1. Synthesis of POSS-Porphyrin Derivatives

**Scheme 1:**
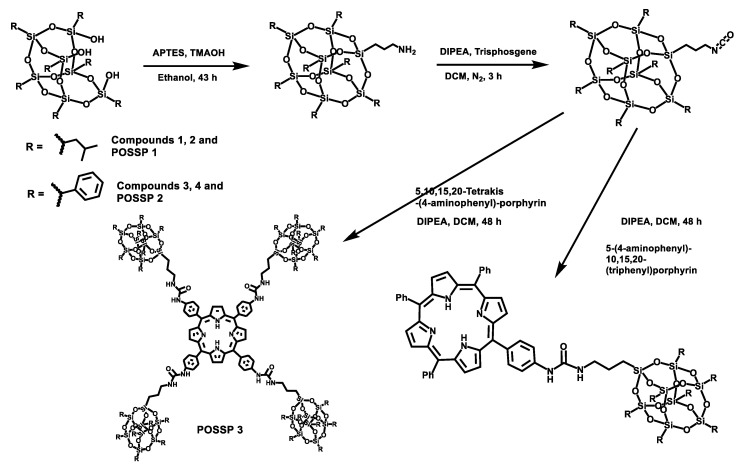
Synthetic scheme for the synthesis of **POSSPs 1**–**3**. The synthesis of **POSSPs 1** and **2** follow a similar approach. First, heptaisobutyl- or heptapheyl-trisilanol POSS was functionalized with aminopropyl trimethoxysilane under basic conditions, followed by transforming the amine to a cyanate group using triphosgene. Finally, 5-(4-aminophenyl)-10,15,20(triphenyl)porphyrin reacted with either the isobutyl (compound **2**) or the phenyl (compound **4**) version of POSS cyanate derivative to afford **POSSP 1** or **2**, respectively. The synthesis of **POSSP 3** was carried out by reacting compound **2** with 5,10,15,20-tetrakis(4-aminophenyl)porphyrin.

POSS contains a wide variety of chemical properties that allow multiple functionalization [22,23]. In this work, POSS-porphyrin molecules (POSSP) were designed to contain different functional groups such as alkyl, aromatic, amino and ammonium, (Figure 1). The synthesis of the POSS-porphyrin derivatives containing hydrophobic groups, isobutyl (**POSSP 1**) and phenyl (**POSSP 2**), was carried out through a multi-step approach (Scheme 1). First, commercially available heptaisobutyl- or heptaphenyl-trisilanol POSS was functionalized with aminopropyl trimethoxysilane via a corner capping reaction under basic conditions to obtain compounds **1** and **3** [22]. The amine group on these compounds were transformed to an isocyanate group by using triphosgene to produce molecules **2** and **4**. The successful formation of the cyanate group was shown by the FT-IR stretching vibration at 2273 cm^−1^. Finally, mono-aminophenyltriphenyl porphyrin was reacted with either compound **2** or **4** via the formation of a urea bond to obtain **POSSP 1** or **2**, respectively. We also synthesized a tetra-substituted POSS-porphyrin (**POSSP 3**) by reacting an excess of compound **2** with tetra-aminophenylporphyrin following the same reaction as described above. The spectroscopic characterization of **POSSPs 1**–**3** demonstrated the successful synthesis of these compounds. Characteristic vibrations in the FT-IR spectra showed the presence of the urea bond at ~1650 cm^−1^ and the Si-O-Si framework at ~1084, 966 and 700 cm^−1^, which are characteristic of the siloxane cage. Confirmation for the fabrication of these POSSP molecules was obtained by ^29^Si NMR with diagnostic signals at −67.0, −67.1 and −67.3 ppm for **POSSPs 1** and **3**; and at −65.3, −77.8 and −78.1 ppm for **POSSP 2**. In addition, MALDI-TOF mass spectro−metry was used to further corroborate the synthesis of the actual POSSP products, showing the expected molecular ions at [M − H]^+^ = 1527.55, [M − 3H]^+^ = 1665.24 and [M − 4H]^+^ = 4267.39, for **POSSPs 1**–**3**, respectively. The complete description for the synthesis and characterization for the intermediate POSS molecules **1**–**4** and **POSSPs 1**–**3** is provided in the Supplementary Materials.

**Scheme 2:**
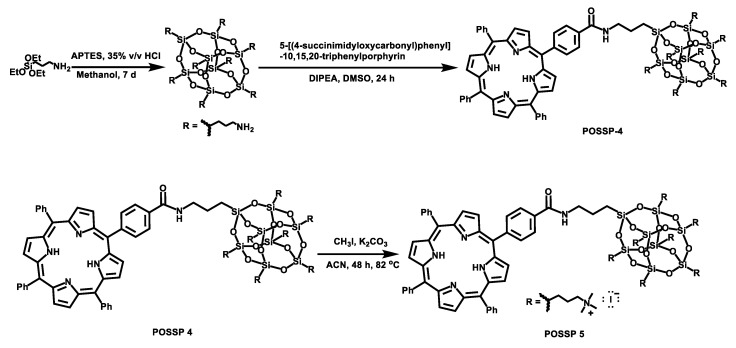
Synthetic scheme for the synthesis of **POSSPs 4** and **5**. First, octaaminopropyl-POSS (compound **5**) was synthesized using aminopropyltriethoxy silane (APTES). This compound was reacted with compound **7** to afford **POSSP 4**. **POSSP 5** was obtained after the methylation of **POSSP 4**.

To synthesize the POSSP molecules containing hydrophilic groups, amine (**POSSP 4**) and trimethylammonium (**POSSP 5**), a different synthetic approach was utilized (Scheme 2). Here, octaaminopropyl-POSS (compound **5**) was synthesized via the hydrolytic condensation approach of aminopropyltriethoxy silane (APTES) [24]. The synthesis of **POSSP 4** was performed through an acyl nucleophilic addition of the amine group in molecule **5** to the activated carboxylic acid in compound **7** followed by purification using column chromatography. Compounds **6** and **7** were synthesized following a procedure reported in the literature; the experimental details and spectroscopic characterization are described in the Supplementary Materials [25]. The synthesis of **POSSP 5** was afforded via a methylation reaction of **POSSP 4** using methyl iodide in the presence of potassium carbonate [11]. The characteristic vibrational frequencies for the amide bond at 1658–1660 cm^−1^, the Si-O-Si framework 1100–1000 and 950–850 cm^−1^ and the Si-C bond at 822 and 758 cm^−1^ were observed. ^29^Si NMR was used to confirm the integrity of the cage; three diagnostic signals at −61.2, −70.7 and −70.9 ppm were determined. The complete description for the synthesis and characterization for the intermediate compounds **5**–**7** and **POSSPs 4** and **5** is provided in the Supplementary Materials.

### 2.2. Spectroscopic Characterization 

**Figure 2:**
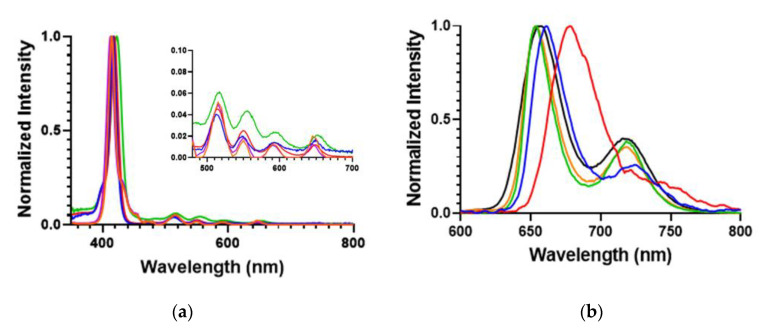
(**a**) Normalized absorption; and (**b**) emission spectra for 6.6 μM solutions of **POSSPs 1** (blue), **2** (red) and **3** (green) in THF and 100 μM solutions of **POSSPs 4** (purple) and **5** (orange) in DMSO. The four Q absorption bands are shown in the inset.

The UV–Vis and fluorescence spectra of the POSSPs was measured and compared with the parent porphyrins. Normalized absorption spectra of the **POSSP 1**–**3** solutions in tetrahydrofuran (THF), and **POSSP 4** and **5** solutions in dimethyl sulfoxide (DMSO) showed the typical Soret and Q-bands for porphyrins in the ranges of 410–420, 510–530, 545–565, 585–595 and 645–660 nm (Figure 2a). The Soret band wavelengths and the corresponding extinction coefficient values are presented in Table 1. The steady-state fluorescence emission spectra with normalized intensities showed two characteristic emission peaks for free-base porphyrins in the ranges of 650–660 and 715–720 nm (Figure 2b and Figure S2). The specific emission wavelengths for the POSSPs are provided in Table 1. The S- and Q-bands of **POSSPs 1**–**3** are slightly blue-shifted with respect to their parent porphyrin, ATPP or TAPP (Table S1 and Figure S1), most likely due to the change in the electron-donating effect of the nitrogen substituent in the para (4-phenyl) position when it is chemically transformed from an amine to an urea group, as has been reported in the literature [26]. On the contrary, the S- and Q-band for **POSSPs 4** and **5** are slightly red-shifted in comparison to compound **6** (Table S1 and Figure S1).

The fluorescence quantum yields (Φ_F_) were determined to indirectly characterize the efficiency with which the POSSP compounds undergo intersystem crossing (ISC) from the excited state to the triplet state, an essential step in ROS generation [27]. Porphyrin derivatives typically generate low Φ_F_, indicating that the majority of photons absorbed by porphyrins undergo ISC to the excited triplet. Φ_F_ in THF for **POSSPs 1**–**3** and in DMSO for **POSSPs 4** and **5** were calculated relative to tetraphenylporphyrin (TPP) in benzene. The data show that the **POSSPs 1**–**3** have lower Φ_F_ values compared to the parent porphyrins (Table 1 and Table S1), as a possible indication of a more efficient ISC. In the case of **POSSPs 4** and **5**, no differences in the Φ_F_ values were observed.

The ^1^O_2_ quantum yield (Φ_Δ_) of the POSSP compounds in dimethyl formamide (DMF) was indirectly determined using 9,10-dimethylanthracene (DMA) as ^1^O_2_ probe. DMA reacts with ^1^O_2_, undergoing a 1,4-cycloaddition that is detected as a decrease in the intensity of the DMA absorption band at 379 nm. The Φ_Δ_ was calculated relative to the reference TPP (Φ_Δ_ = 0.62) [28]. using the slope of the time-dependent decomposition of DMA plots (Ln([DMA_0_]/[DMA]) versus irradiation times (Figure S3) and Equation (1) [28].

Φ_F,Sample_ = Φ_F,Reference_ (m_Sample_/m_Rerefence_) (η^2^_Sample_/η^2^_Rerefence_) (1)

where Φ_F,Reference_ represents the fluorescence quantum yield of a fluorophore reference (TPP), m is the slope of the plotted data relative to the area of the emission peak against the absorption of the fluorophore and η is the refractive index [29].

The experimental protocol was validated by comparing tetrahydroxy-phenyl-porphyrin and tetraamino-phenyl-porphyrin molecules with known Φ_Δ_ in DMF (Φ_Δ_ = 0.57 ± 0.03 and Φ_Δ_ = 0.58 ± 004) [30]. The measured quantum yield values matched the literature values within ±3% error, with a Φ_Δ_ values of 0.59 ± 0.01 and 0.58 ± 0.01 for tetrahydroxy-phenyl-porphyrin and tetraamino-phenyl-porphyrin molecules, respectively. 

The Φ_Δ_ values obtained for **POSSPs 1**–**5** are shown in Table 1. Interestingly, all the Φ_Δ_ values for the POSSP compounds containing hydrophobic groups **POSSPs 1**–**3** are higher than those corresponding to the parent porphyrins (*p* < 0.0001, Table S1). **POSSPs 1**–**3** showed an increase in the Φ_Δ_ values by 82%, 33% and 19%, respectively. In the case of the POSSPs with hydrophilic groups, only the Φ_Δ_ value for **POSSP 4** was higher than the compound **6** (*p* < 0.001, Table S1) with an increase of 9%. Hence, the Φ_Δ_ values for the POSSPs follow this trend **POSSP 1** > **POSSP 3** ≈ **POSSP 4** > **POSSP 5** > **POSSP 2**. To confirm that this increase is indeed associated with the POSSP molecules and not due to the effect of physical mixture of POSS and the parent porphyrin in solution, the Φ_Δ_ values for the physical mixture of compound **2** + ATPP and compounds **5**/**6** were determined. As shown in Table S1, the Φ_Δ_ values for the mixtures are lower than the corresponding **POSSP 1** or **4** (*p* < 0.0001), which confirms that the enhancement in Φ_Δ_ is intrinsic to the POSSPs. The Φ_Δ_ value could depend on the following factors: (i) triplet state properties, including quantum yield, lifetime and energy; (ii) the ability of substituents to quench ^1^O_2_; and (iii) the efficiency of energy transfer from the excited triplet state to ground state molecular oxygen [31]. In the case of porphyrins, the triplet state after irradiation could be inhibited by mutual energy transfer since these molecules favor π-stacking, also known as self-quenching effect [32]. This effect results in a decline of the ability of porphyrins to generate ^1^O_2_. However, due to the unique 3D structure of POSS, its steric hindrance suppresses self-quenching of the excited states of porphyrins resulting in the observed enhancement of Φ_Δ_ for POSSPs [17,18]. It is also relevant to point out that the increase in the Φ_Δ_ values for the POSSPs may have a major impact on their PDT performance.

### 2.3. Photodynamic Therapy of Triple-Negative Breast Cancer Using POSS-Porphyrin Molecules 

**Figure 3:**
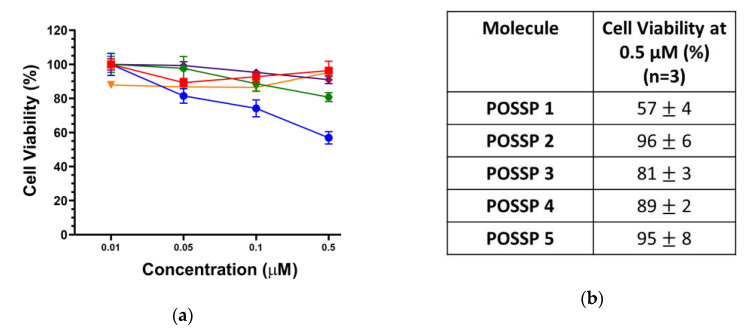
(**a**) Dose–response plot for the phototoxicity of **POSSPs 1** (blue), **2** (red), **3** (green), **4** (purple) and **5** (orange) in MDA-MB-231 cells. (**b**) The table shows the cell viability at 0.5 µM of POSSPs.

Triple-negative breast cancer (TNBC) subtype is characterized by the lack of targetable markers, which accounts for about 10–20% of the newly diagnosed breast cancer cases [33]. Approximately 50% of the patients diagnosed with early-stage TNBC experience recurrence, and 37% die within the first five years after surgery [34]. As TNBC lacks targetable receptors, patients with TNBC are treated with conventional chemotherapy and radiotherapy, which are associated with toxic side effects and development of resistance leading to aggressive relapse and distant metastasis [35]. PDT has been used to treat different types of cancer such as those originated in the skin, head and neck, breast and lung [2,36]. Recently, PDT has been explored as a promising alternative to treat TNBC [6,8,37,38]. In this work, the cytotoxicity and phototoxicity of POSSPs in a tripe negative breast cancer cell line (MDA-MB-231) were evaluated using the MTS assay. As expected, no major cytotoxicity was observed in the range of concentration tested in the absence of light (Figure S4). Photosensitizers are only toxic through the generation of reactive oxygen species after excitation with light and in the presence of molecular oxygen [3]. To compare the PDT performance of all the POSSPs developed in this work, due the hydrophobicity of **POSSP 1**–**3**, a non-toxic amount of DMSO was used to carry out these experiments. As shown in Figure 3, at the highest concentration evaluated (0.5 µM), **POSSP 1** showed a reduction in cell proliferation of 43 ± 4% followed by **POSSP 3** with 19 ± 3% and **POSSP 4** with 11 ± 2%. Interestingly, this trend in cell phototoxicity, **POSSP 1** > **POSSP 3** > **POSSP 4**, follows a similar trend as the Φ_Δ_ values. This corroborates the importance of the singlet oxygen generation for the PDT effect. **POSSPs 2** and **5** did not show significant phototoxicity at the highest concentration.

**Figure 4:**
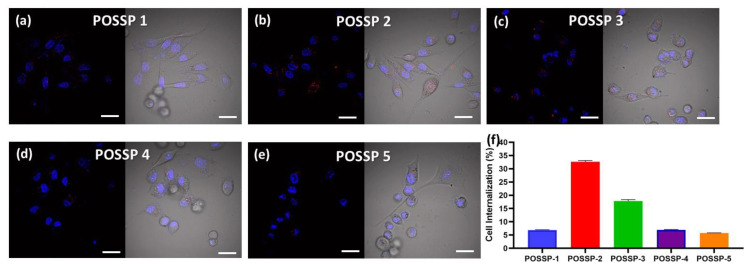
Confocal micrographs of MDA-MB-231 cells inoculated with **POSSPs 1**–**5**, respectively (**a**–**e**). The cell nuclei are observed in the blue channel after staining with Hoechst 33342. The fluorescence in the red channel shows the presence of POSSPs. The merged micrographs show the of POSSPs inside MDA-MD-231 cells. Scale bar, 30 μm. The internalization of **POSSPs 1** (blue), **2** (red), **3** (green), **4** (purple) and **5** (orange) in MDA-MB-231 cells was quantified using flow cytometry (**f**).

To investigate the interaction of the POSSPs with cells, we used confocal microscopy. MDA-MB-231 cells were incubated for 24 h in the presence of POSSPs (0.5 µM). Fluorescence associated with porphyrins was observed in the range of 600–700 nm (Red-channel). Moreover, cell nuclei were DAPI-stained and observed in the range of 500–550 nm (Blue-channel) (Figure S5). The merged image between both channels showed the presence of POSSPs (Figure 4a–e). It is clear that the number of red spots follow the trend: **POSSP 2** > **POSSP 3** > **POSSP 1** > **POSSP 4** ≈ **POSSP 5**. The overlapped image and the corresponding differential interference contrast (DIC) micrograph allowed us to confirm the presence of POSSPs within the cell bodies of MDA-MB-231 cells (Figure 4a–e). To corroborate these observations, we used flow cytometry. MDA-MB-231 cells were inoculated with **POSSPs 1**–**5** at a concentration of 0.5 µM for 24 h. The flow cytometry data show that **POSSPs 2** and **3** are indeed internalized in a much higher percentage than the other POSSPs. Interestingly, these two POSSPs that were designed with hydrophobic properties did show higher uptake by cells; however, that was not the case for **POSSP 1**. One approach to indirectly evaluate the interaction of porphyrins with cells is by determining the partition coefficients, which is the ratio of the concentration of a compound in a biphasic organic/aqueous system. Here, we determined the *n*-octanol/water partition coefficients (log P_OW_) and used the obtain values to calculate *n*-butanol/water partition coefficients (log P_OW_) (Table S2) [39,40]. **POSSPs 1** and **2** show positive values of log P_OW_ as an indication of their affinity toward lipid phases. Nevertheless, these values were lower than the parent porphyrins as an indication that the POSS cage added hydrophilic character to the POSSPs. This is confirmed with **POSSP 3**, which present the lowest negative log P_OW_ value for the series of POSSPs. Negative log P_OW_ values for **POSSPs 4** and **5** were also determined, which indicates that the compounds are slightly hydrophilic. In the case of functionalized porphyrins, previous reports have shown that the log P_OW_ can be associated with their interaction with cells; in particular, the cellular uptake increased proportionally with their partition coefficients [32]. Moreover, the phototoxicity of functionalized porphyrins in cancer cells follow a trend with more lipophilic compounds being more phototoxic than the hydrophilic ones [41]. In our study, **POSSP 1** which has a larger value of log P_OW_ shows increased PDT effect; nevertheless, the internalization according to confocal and flow cytometry data is lower than the other two hydrophobic POSSPs. It seems that, in this case, the photochemical properties of the POSSPs, in particular ^1^O_2_ generation, play a major role in the PDT outcome. This hypothesis is supported by the results obtained with the porphyrin control (ATPP) where a low Φ_Δ_ value was obtained, but a high internalization in MDA-MB-231 cells was observed, and minimal PDT effect was obtained (Figure S6), most likely because a low Φ_Δ_ value was obtained (Table S1). Nevertheless, it is important not to rule out other factors such as the subcellular localization of these compounds.

**Figure 5:**
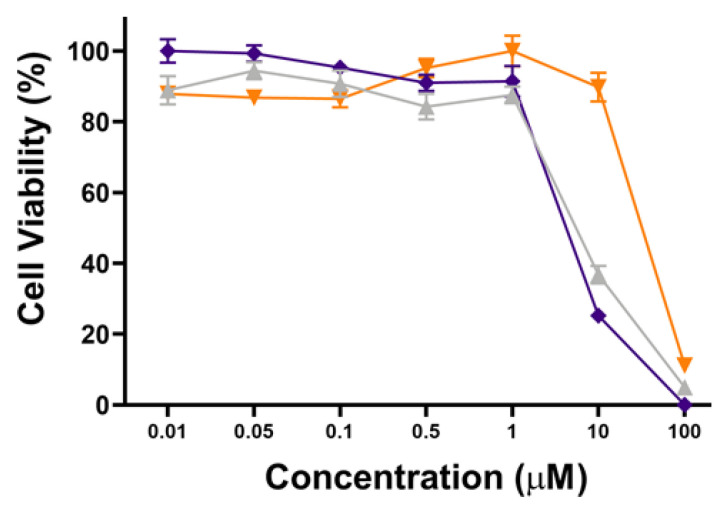
Dose–response plot for the phototoxicity of **POSSPs 4** (purple) and **5** (orange) and compound 6 (light gray) in MDA-MB-231 cells.

To further show the PDT performance of hydrophilic POSS, the phototoxicity and cytotoxicity of **POSSPs 4** and **5** in MDA-MB-231 cells were evaluated at higher concentrations. Figure 5 shows that **POSSP 4** has a similar concentration-dependent phototoxicity profile as the porphyrin control. The IC_50_ values calculated for **POSSP 4** and compound **6** were 6.2 and 7.3 µM, respectively. In the case of **POSSP 5**, the structural properties of the molecule also play a role in its toxicity (Figure S7). The toxicity is associated with the amphiphilic character of **POSSP 5**, and the permanent positive charge that most likely disrupt the cell membrane of MDA-MB-231 cells, producing an effect similar to a surfactant.

## 3. Materials and Methods 

### 3.1. Materials

All commercial solvents were of reagent grade or higher and used as received. All experiments with moisture/air-sensitivity were performed in anhydrous solvents under a nitrogen atmosphere. Column chromatography was performed using silica G60 (70–230 mesh) (SiliCycle Inc., Quebec City, Quebec, Canada). Trisilanol hepta(isobutyl) POSS and trisilanol hepta(phenyl) POSS were purchased from Hybrid Plastics Inc (Hattiesburg, MS, USA). (3-aminopropyl) triethoxysilane (APTES) were purchased from Alfa Aesar (Haverhill, MA, USA). Pentahydrate tetramethyl ammonium hydroxide in methanol (TMAOH), *N*,*N*’-diisopropylethylamine, triphosgene, pyrrole, benzaldehyde, 4-formylbenzoic acid, boron trifluoride etherate (BF_3_∙Et_2_O), *p*-chloranil, *N*-hydroxysuccinimide (NHS), N,N’-dicyclohexylcarbodiimide (DCC), 4-dimethylaminopyridine (DMAP), iodomethane and dimethylanthracence (DMA) were purchased from Sigma-Aldrich (St. Louis, MO, USA). 5-(4-aminophenyl)-10,15,20-(triphenyl)porphyrin (ATPP), 5,10,15,20-tetra(4-aminophenyl) porphyrin (TAPP) and 5,10,15,20-(tetraphenyl)porphyrin (TPP) were purchased from Frontier Scientific (Logan, UT, USA). Potassium carbonate was purchased from EMD Millipore (Burlington, MA, USA). Sterile-filtered DMSO was used for all cell experiments and purchased from Sigma-Aldrich (St. Louis, MO, USA). Roswell Park Memorial Institute (RPMI 1640), fetal bovine serum (FBS), penicillin–streptomycin (pen-strep), phosphate buffer saline (PBS, 1X) and trypsin were purchased from Corning (Corning, NY, USA). CellTiter 96^®^ AQueous Assay was obtained from Promega (Madison, WI, USA). 

NMR spectra were recorded at room temperature in CDCl_3_ and DMSO-D_6_ solvents using a 300 MHz or 500 MHz JEOL NMR spectrometer (Peabody, MA, USA). Mass spectra were collected by a Voyager Biospectrometry Laser MALDI-TOF and a spectrometer thermal Scientific MSQ Plus ESI spectrometer (Waltham, MA, USA). Infrared spectra of liquid and solid samples were collected on a Perkin-Elmer 100 IR spectrophotometer from 4000 to 700 cm^−1^ (Waltham, MA, USA). Steady-state absorption and fluorescence spectra were recorded on a Varian Cary Bio50 UV-Vis absorption spectrophotometer and a Cary Eclipse fluorescence spectrophotometer, respectively (Varian, Sidney, Australia). Photophysical properties were measured using a RF-5301 PC spectrofluorophotometer Shimadzu (Kyoto, Japan). A homemade LED device emitting at 630 nm (24.5 mW/cm^2^) was used for the in vitro experiments (Laboratory of Technological Support, São Carlos Institute of Physics, São Carlos, Brazil).

### 3.2. Photophysical Characterization 

#### 3.2.1. UV–Vis/Fluorescence Spectroscopy 

The UV–Vis spectra were recorded from 300 to 800 nm using solutions of **POSSPs 1**–**3** in THF (6.6 µM) and **POSSPs 4** and **5** in DMSO (100 µM) in quartz cuvettes (1 cm path length). Similar conditions were used for the parent porphyrins (TPP, ATPP, TAPP and compound **6**). The fluorescence spectra for POSSPs and parent porphyrins were obtained in the same solutions described above using an excitation wavelength of 520 nm. The fluorescence spectra were recorded from 600 to 800 nm. 

The extinction coefficients were obtained using the Beer’s Law equation from the linear regression of absorption values vs. concentration. For **POSSPs 1**–**3**, several concentrations in THF ranging from 0.1 to 10 µM were used. In the case of **POSSPs 4** and **5**, several concentrations in DMSO ranging from 0.1 to 10 µM were utilized. Similar conditions were used for the parent porphyrins (TPP, ATPP, TAPP and compound **6**).

#### 3.2.2. Fluorescence Quantum Yield 

The fluorescence quantum yields for air-saturated solutions (Φ_F_) in THF (**POSSPs 1**–**3**) or DMSO (**POSSPs 4** and **5**) were determined using the comparative method. TPP was used as a reference with a fluorescence quantum yield of 0.12 in benzene [42]. The POSSPs concentrations ranged from 0.1 to 10 µM (THF) for **POSSPs 1**–**3** and from 3 to 15 µM (DMSO) for **POSSPs 4** and **5**. The excitation wavelength was 520 nm and the excitation and emission slit width were 2 nm. The fluorescence quantum yields were measured according to the comparative method described by Equation (1).

#### 3.2.3. Singlet Oxygen Quantum Yield 

The ^1^O_2_ quantum yields (Φ_Δ_) were determined through an indirect method using dimethylanthracence (DMA) as the singlet oxygen probe. Several solutions containing DMF were air saturated and prepared with DMA (50 μM) and the POSSPs or parent porphyrins (TPP, ATPP, TAPP and compound **6**) (5 μM). These solutions were covered with aluminum foil to avoid any premature quenching. Quartz cuvettes (1 cm × 1 cm) were filled with 1 mL of the solution, placed in a spectrofluorophotometer (xenon lamp, Shimadzu RF-5301 PC) and irradiated at 515 nm for 600 s. The absorbance decay of DMA was monitored at 380 nm, which was corrected from light scattering by subtracting the spectra of POSSPs. The Φ_Δ_ was calculated using Equation (2).

Φ_∆,S_ = Φ_∆,R_ (m_Sample_/m_Rerefence_) (1−10^−absReference^/1−10^−absSample^) (2)

where Φ_∆,S_ is the singlet oxygen quantum yield of the sample and m is the slope of the plotted data relative to the area of the emission peak against the absorption of the reference [42].

### 3.3. Partition Coefficient (log P_OW_)

The log P_OW_ values of the POSSPs and controls were first measured in an *n*-butanol/water mixture, and then correlated with corresponding values in *n*-octanol/water using a calibration curve reported in the literature [39,40]. Distilled water and butanol were thoroughly mixed for 24 h at 25 °C to achieve solvent saturation in both phases. Then, the porphyrin derivatives were added to the mixture and the phases separated through a separatory funnel. The UV–Vis absorbance of the compounds in water and n-butanol was recorded at 420 nm.

logP_BW_ = log[(A_b_/A_w_)(V_w_/V_b_)] (3)

where A_b_ is the absorbance of *n*-butanol phase; A_W_ is the absorbance of water phase; V_W_ is the volume of water; and V_b_ is the volume of n-butanol. The partition coefficient of porphyrin derivatives in *n*-butanol/water was obtained using Equation (3). The log P_OW_ values were calculated using Equation (4) [39].

logP_OW_ = −0.54 + 1.55 log(P_BW_) (4)

### 3.4. Cell Culture

MDA-MB-231, a human invasive TNBC cell line, was purchased from American Type Culture Collection (ATCC). Breast cancer cells were cultured in RPMI 1640 medium supplemented with 10% FBS and 1% pen-strep at 37 °C with 5% CO_2_ atmosphere. The culture media was changed every other day. All cell cultures were maintained in 25 or 75 cm^2^ cell culture flasks and the cells were passaged at 70–80% confluency every 2–4 days.

### 3.5. In Vitro Cyto- and Phototoxicity 

The phototoxicities of ATPP, TAPP, compound **6** and **POSSPs 1**–**5** were tested by using the MTS assay. For this study, MDA-MB-231 cells were seeded in a 96-well plate at a density of 5 × 10^3^ cells per well in 100 µL of complete media and incubated at 37 °C in 5% CO_2_ atmosphere for 24 h. After removing the cell culture medium, **POSSPs 1**–**5** at different concentrations (0.01–0.5 µM) were prepared in cell media from a stock solution in DMSO with a final volume of the organic solvent not larger than 1% vol. After 48 h of incubation in the presence of POSSPs, the culture media was removed, and the cells were washed twice with phosphate buffer solution. MDA-MB-231 cells were illuminated with the red light (630 nm) at a fluence rate of 24.5 mW/cm^2^ for 20 min. Control experiments were maintained in the same conditions, but in the dark for the same interval of time. After irradiation, the media was replaced by fresh media and the cells were allowed to grow for an additional 24 h. The cyto- and phototoxicities of compound **6** and **POSSPs 4** and **5** were also evaluated following the above described protocol, but using concentrations ranging 0.01–100 µM.

To quantify the phototoxic or dark toxicity effects of the experiments above described, the treated MDA-MB-231 cells were subjected to cell viability assay using the CellTiter 96^®^ Aqueous solution assay. To perform the assay, the cell media was removed, and the cells were washed once with phosphate buffer solution. Fresh media (100 µL) together with 20 µL of CellTiter 96^®^ was added into each well and incubated for 2–3 h at 37 °C in 5% CO_2_ atmosphere. Cell viability (%) was calculated as follows: viability = (A_sample_/A_control_) × 100%, where A_sample_ and A_control_ denote absorbance values of the sample and control wells measured at 490 nm, respectively. The results are reported as the average ± SD of three experiments. The IC_50_ values were determined using GraphPad Prism (v8.1.2 for macOS, La Jolla California, CA, USA) fitting the viability data to a sigmoidal curve mathematical model.

### 3.6. Flow Cytometry

Six-well plates were prepared with 1 × 10^5^ MDA-MB-231 cells per well in complete RPMI media (2 mL) and incubated at 37 °C in 5% CO_2_ for 48 h. The media was removed from each well and media solutions of control porphyrin (ATPP) and **POSSPs 1**–**5** (2 mL, 0.5 µM) were added to the wells. The cells were incubated at 37 °C in 5% CO_2_ atmosphere for 24 h. The cell media was removed from the wells, and the cells were washed with phosphate buffer solution two times. The cells were incubated for 3–5 min in the presence of trypsin (500 µL). Cells were removed and transferred to a centrifuge tube. Media (500 µL) was added to each well to remove any remaining cells and mixed with the previous solution. The cell pellet was obtained after centrifugation (2.5k RPM, 10 min). The supernatant was discarded, and phosphate buffer solution (500 µL) was added to each tube. The cell pellet was mixed and transferred to flow cytometry tubes. The cell internalization of POSSP compounds was measured as the percentage of positive cells using a flow cytometer (BD LSRFortessa).

### 3.7. Confocal Microscopy

Six-well plates containing a cover glass were prepared with 5 × 10^4^ MDA-MB-231 cells per well in complete RPMI media (2 mL) and incubated at 37 °C in 5% CO_2_ for 48 h. The media was removed from each well and media solutions of **POSSPs 1**–**5** (2 mL, 0.5 µM) were added to the wells. The cells were incubated at 37 °C in 5% CO_2_ atmosphere for 24 h. The cell media was removed from the wells, and the cells were washed with phosphate buffer solution two times. The cell nuclei were stained with Hoechst 33,342 for 15 min. All microscopy images were acquired with an Olympus Fluoview FV 1000 CLSM.

### 3.8. Statistical Analysis

All data in the manuscript are presented as mean ± SD unless mentioned otherwise. To compare Φ_Δ_ values, the statistical analysis was performed with one-way ANOVA using Tukey’s multiple comparison test. To calculate the singlet oxygen quantum yields (n = 3) and IC_50_ values (n = 6) for the cell viability studies, GraphPad prism software was used. Cellular uptake using flow cytometry was evaluated with a minimum of 5000 gated cells. All the statistical analyses were performed using GraphPad Prism (v8.2.0 for Windows) with α = 0.05 and reported as asterisks assigned to the *p*-values: **** *p* ≤ 0.0001, *** *p* ≤ 0.001, ** *p* ≤ 0.01, * *p* ≤ 0.05 and ns *p* > 0.05.

## 4. Conclusions

A series of polyhedral oligomeric silsesquioxane porphyrin derivatives was synthesized and characterized. The effect of the POSS substituents on the photochemical properties of the POSSPs showed that hydrophobic groups produced an increase on the singlet oxygen quantum yield as compared with their parent porphyrins. Moreover, a better phototoxic efficacy was achieved with these molecules at lower concentrations. POSSPs with hydrophilic substituents presented an improved PDT effect at higher concentrations in MDA-MB-231 cells. We envision that, by using POSS as a scaffold for functionalization of photosensitizers, the outcome of PDT against cancer can be improved.

## Figures and Tables

**Table 1:** Photophysical and photochemical properties of POSSPs.

Sample	λ_Soret_ (nm) [ε × 10^3 (^M^−1^ cm^−1^)] (n = 3)	λ_Emission_ ( nm)	Φ_Δ_ (n = 3)	Φ_F_ (n = 3)
**POSSP 1**	416, 22.1 ± 8.3	653, 720	0.82 ± 0.01	0.14 ± 0.01
**POSSP 2**	419, 46.2 ± 9.0	653, 719	0.60 ± 0.02	0.13 ± 0.01
**POSSP 3**	422, 117.3 ± 34.4	663,719	0.70 ± 0.01	0.13 ± 0.02
**POSSP 4**	417, 2.7 ± 0.1	648,712	0.70 ± 0.01	0.15 ± 0.01
**POSSP 5**	420, 2.6 ± 0.2	647,711	0.66 ± 0.01	0.10 ± 0.01

## Supplementary Materials

The following are available online. Synthesis and characterization of POSSPs molecules, UV–Vis and fluorescence spectra of porphyrin controls, time-dependent decomposition of DMA plots, cell viability graphs of POSSPs and porphyrin controls, photophysical and photochemical properties of porphyrin controls and partition coefficients.
