# Supplementary material for: Evaluation of Polyhedral Oligomeric Silsesquioxane Porphyrin Derivatives on Photodynamic Therapy
Source: Molecules. 2020 Oct 27;25(21):4965. doi: 10.3390/molecules25214965 (PMC7662523; doi:10.3390/molecules25214965)
Supplement: Supplementary file 1 [file molecules-25-04965-s001.pdf]

Supplementary Materials

Evaluation of Polyhedral Oligomeric Silsesquioxane Porphyrin Derivatives on Photodynamic Therapy

Paolo Siano‡[1], Alexis Johnston‡[1], Paula Loman-Cortes[1,2], Zaneta Zhin[1] and Juan Vivero-Escoto[1,3]*

[1]Department of Chemistry, University of North Carolina Charlotte, Charlotte NC 28223, USA

[2]Nanoscale Science Program, University of North Carolina Charlotte, Charlotte NC 28223, USA

[3]Center for Biomedical Engineering and Science, University of North Carolina Charlotte, Charlotte NC 28223, USA

Materials and Methods

Synthesis and characterization of POSSs molecules

Synthesis of Isobutyl-POSS-Porphyrin (POSSP-1)

Synthesis of Aminopropyl hepta(isobutyl) POSS (1-(3-amino)propyl-3,5,7,9,11,13,15-heptaisobutyl pentacyclo[9.5.1.1(3,9).1(5,15).1(7,13)]octasiloxane) **(1)**

The synthesis of compound **1** was carried out as previously reported with slight modifications [1]. Trisilanol hepta(isobutyl) POSS (2.00 g, 2.52 mmol) was dispersed in ethanol (12.5 mL) under stirring (550 rpm) followed by the addition of APTES (424 μL, 435 mg, 1.97 mmol) and TMAOH (40 μL, 0.06 mmol, 25% w/v). The mixture was stirred (700 rpm) for 43 h at 40°C. A significant amount of aminopropyl hepta(isobutyl) POSS precipitated during the reaction time. The dispersion was centrifuged and the supernatant discarded. The solid product was washed twice with acetonitrile. The product was dried under vacuum for 48 h (Yield = 84% wt).

^{1}H-NMR (300 MHz, $CDCl_3$, ppm) δ: 2.67 (t, 2H, -C*H*$_2$-N-), 1.85 (m, 7H, -C*H*-), 1.52 (m, 2H, -C*H*$_2$-), 0.94 (d, 42H, C*H*$_3$), 0.60 (m, 16H, -Si-C*H*$_2$-); ^{29}Si-NMR (500 MHz, $CDCl_3$, ppm) δ: -66.8, -67.2, -67.4. FTIR (cm^{-1}): 2953, 2906 and 2870 (C-H), 1600 (N-H), 1465 (C-N), 1228 (Si-C), 1081 (Si-O-Si), 955 (Si-O-Si), 740 (Si-C). MALDI-TOF (m/z): $[M]^+$ = 873.01 observed; $[M]^+$ = 873.31 calcd.

Synthesis of isocyanato propyl hepta(isobutyl) POSS (1-isocyanatopropyl-3,5,7,9,11,13,15-heptaisobutyl pentacyclo[9.5.1.1(3,9).1(5,15).1(7,13)]octasiloxane) (2)

The synthesis of compound **2** was carried out as previously reported with slight modifications [2]. Compound **1** (150 mg, 0.172 mmol) was dissolved in dry dichloromethane (2 mL) at room temperature, and the solution was stirred slowly (300 rpm) under nitrogen atmosphere. DIPEA (60 µL, 44.5 mg, 0.34 mmol) and triphosgene (25.5 mg, 0.086 mmol) were added to this solution under slow stirring and N_2 atmosphere. After 3 h, the stirring speed and the nitrogen flow were increased to cause the evaporation of the solvent (evaporation time: 10 min). The product was washed twice with acetonitrile. Compound **2** was dried under vacuum for 48 h (Yield = 71% wt).

^{1}H-NMR (300 MHz, $CDCl_3$, ppm) δ: 3.28 (t, 2H, -C*H*$_2$-N=C=O), 1.85 (m, 7H, -C*H*-), 1.71 (m, 2H, -C*H*$_2$-), 0.94 (d, 42H, C*H*$_3$), 0.66 (t, 2H, -Si -C*H*$_2$-), 0.60 (m, 14H, -Si -C*H*$_2$-); ^{29}Si-NMR (500 MHz, $CDCl_3$ ppm) δ: -67.1, -67.4, -67.7. FTIR (cm^{-1}): 2954 and 2870 (C-H), 2273 (N=C=O), 1465 (C-N), 1229 (Si-C), 1084 (Si-O-Si), 955 (Si-O-Si), 739 (Si-C).

Synthesis of hepta(isobutyl)-POSS-Porphyrin (5-(4-[3-(3-(3,5,7,9,11,13,15-heptaisobutyl pentacyclo[9.5.1.1(3,9).1(5,15).1(7,13)]octasiloxane) propyl) ureido] phenyl)-10,15,20-(triphenyl)porphyrin) (POSSP-1)

Compound **2** (20 mg, 0.022 mmol) was dissolved in dry dichloromethane (2 mL) at room temperature. To this solution, 5-(4-aminophenyl)-10,15,20-(triphenyl)porphyrin (ATPP) (28 mg, 0.045 mmol) and excess DIPEA were added. The final solution was stirred at room temperature for 48 h in a sealed flask. The product was separated using column chromatography on silica gel (DCM:MeOH; 99:1). After purification, **POSSP-1** was dried and obtained as a dark powder (Yield = 82 % wt).

^{1}H-NMR (300 MHz, $CDCl_3$, ppm) δ: 8.87 (m, 8H, Py-H), 8.18 (m, 8H, Ph-H), 7.74 (m, 11H, Ph-H), 3.34 (t, 2H, -C*H*$_2$-N-), 1.88 (m, 7H, -C*H*-), 1.76 (m, 2H, -C*H*$_2$-), 0.96 (d, 42H, C*H*$_3$), 0.71 (t, 2H, -Si-C*H*$_2$-), 0.61 (m, 14H, -Si -C*H*$_2$-); ^{29}Si-NMR (500 MHz, $CDCl_3$, ppm) δ: -67.0, -67.1, -67.3. FTIR (cm^{-1}): 3318 (N-H), 2925 and 2870 (C-H), 1655 (C=O), 1465 (C-N), 1227 (Si-C), 1084 (Si-O-Si), 966 (Si-O-Si), 700 (Si-C). MALDI-TOF (m/z): $[M-1]^+$ = 1527.55 observed; $[M]^+$ = 1528.55 calcd.

Synthesis of Phenyl-POSS-Porphyrin (POSSP-2)

Synthesis of Aminopropyl hepta(phenyl) POSS (1-(3-amino)propyl-3,5,7,9,11,13,15-heptaphenyl pentacyclo[9.5.1.1(3,9).1(5,15).1(7,13)]octasiloxane) (3)

The synthesis of compound **3** was carried out as previously reported with slight modifications [3]. Trisilanol hepta(phenyl) POSS (2.48 gr, 2.7 mmol) was dispersed under stirring (500 rpm) in toluene (4.0 mL) and placed in a dry ice/acetone bath at -10 °C for 5 min. To this solution, APTES (616 µL, 600 mg, 2.7 mmol) was added. The final dispersion was allowed to warm up to room temperature under stirring (500 rpm) for 16.5 h. Then, acetonitrile (20 mL) was added to precipitate the product. Finally, the supernatant was removed after centrifugation and the final product was washed twice with acetonitrile. Compound **3** was dried under vacuum for 48 h (Yield = 61% wt).

^{1}H-NMR (300 MHz, $CDCl_3$, ppm) δ: 7.74 (m, 14H, Ph-H), 7.44 (m, 7H, Ph-H), 7.37 (m, 14H, Ph-H), 2.66 (t, 2H, -C*H*$_2$-N-), 1.62 (m, 2H, -C*H*$_2$-), 0.84 (t, 2H, -Si-C*H*$_2$-); ^{29}Si-NMR (500 MHz, $CDCl_3$, ppm) δ: -64.5, -77.8, -78.3. FTIR (cm^{-1}): 3073 (N-H), 3051 (C-H, sp^2), 2921 (C-H, sp^3), 1595 (N-H), 1431 (C-N), 1082 (Si-O-Si), 1027 (Si-O-Si), 780 (Si-C). MALDI-TOF (m/z): $[M]^+$ = 1013.13 observed; $[M]^+$ = 1013.29 calcd.

Synthesis of isocyanato propyl hepta(phenyl) POSS (1-isocyanatopropyl-3,5,7,9,11,13,15-heptaphenyl pentacyclo[9.5.1.1(3,9).1(5,15).1(7,13)]octasiloxane (4)

Compound **3** (174 mg, 0.172 mmol) was dissolved in dry dichloromethane (2 mL) at room temperature and the solution was stirred slowly (300 rpm) under nitrogen atmosphere. Triphosgene (25.5 mg, 0.086 mmol) and DIPEA (60 µL, 44.5 mg, .34 mmol) were added to this solution under slow stirring and N_2 atmosphere. After 3 h, the stirring speed and the nitrogen flow were increased to

cause the evaporation of the solvent (evaporation time: 10 min). The product was washed twice with acetonitrile twice. Compound **4** was dried under vacuum for 48 h (Yield = 78% wt).

^{1}H-NMR (300 MHz, $CDCl_3$, ppm) δ: 7.71 (m, 14H, Ph-H), 7.41 (m, 7H, Ph-H), 7.38 (m, 14H, Ph-H), 3.18 (m, 2H, -CH2-N=C=O), 1.80 (m, 2H, -CH2-), 0.84 (m, 2H, -Si-CH2-); ^{29}Si-NMR (500 MHz, $CDCl_3$, ppm) δ: -65.3, -77.6, -78.1. FTIR (cm^{-1}): 3073 (C-H, sp2), 2933 (C-H, sp^3), 2271 (N=C=O), 1600 (C=O), 1431 (C-N), 1085 (Si-O-Si), 1027 (Si-O-Si), 743 (Si-C).

Synthesis of hepta(phenyl)-POSS-Porphyrin (5-(4-[3-(3-(3,5,7,9,11,13,15-heptaphenyl pentacyclo[9.5.1.1(3,9).1(5,15).1(7,13)]octasiloxane) propyl) ureido] phenyl)-10,15,20-(triphenyl)porphyrin) (POSSP-2)

Compound **4** (22 mg, 0.022 mmol) was dissolved in dry dichloromethane (2 mL) at room temperature. To this solution, 5-(4-aminophenyl)-10,15,20-(triphenyl)porphyrin (ATPP) (28 mg, 0.045 mmol) and excess DIPEA were added. The final solution was stirred at room temperature for 48 h in a sealed flask. The product was separated using column chromatography on silica gel (Toluene:MeOH; 80:1). After purification, compound **6** was dried and obtained as a dark powder (Yield = 73% wt).

^{1}H-NMR (300 MHz, $CDCl_3$, ppm) δ: 8.83 (m, 8H, Py-H), 8.21 (m, 8H, Ph-H), 7.80-7.74 (m, 25H, Ph-H), 7.44 (m, 7H, Ph-H), 7.37 (m, 14H, Ph-H), 3.34 (t, 2H, -CH2-N-), 1.62 (m, 2H, -CH2-), 0.84 (t, 2H, -Si-CH2-); ^{29}Si-NMR (500 MHz, $CDCl_3$, ppm) δ: -76.0, -69.1. FTIR (cm^{-1}): 3645 (-NH-CO-NH), 3380 (-NH-), 3062 (C-H, sp2), 2960 (C-H, sp^3), 1737 (C=O), 1469 (C-N), 1135 (Si-O-Si), 1109 (Si-O-Si), 742 (Si-C). MALDI-TOF (m/z): $[M]^+$ = 1668.24 observed; $[M]^+$ = 1668.33 calcd.

Synthesis of tetra-Isobutyl-POSS-Porphyrin (POSSP-3) (tetra-(4-[3-(3-(3,5,7,9,11,13,15-heptaisobutyl pentacyclo[9.5.1.1(3,9).1(5,15).1(7,13)]octasiloxane) propyl) ureido] phenyl)-10,15,20-(triphenyl)porphyrin)

Compound **2** (120 mg, 0.132 mmol) was dissolved in dry dichloromethane (2 mL) at room temperature. To this solution, 5,10,15,20-tetra(4-aminophenyl)porphyrin (TAPP) (30 mg, 0.045 mmol) and excess DIPEA were added. The final solution was stirred at room temperature for 48 h in a sealed flask. The product was separated using column chromatography on silica gel (DCM:MeOH; 5:1). After purification, compound **9** was dried and obtained as a dark red powder (Yield = 83 % wt).

^{1}H-NMR (300 MHz, $CDCl_3$, ppm) δ: 8.87 (m, 8H, Py-H), 8.05 (m, 8H, PhH),7.80- 7.70 (m, 8H, Ph-H), 3.34 (t, 8H, -CH2-N-), 1.88 (m, 28H, -CH-), 1.76 (m, 8H, -CH2-), 0.94 (d, 168H, CH3), 0.70- 0.60 (m, 64H, -Si -CH2-); ^{29}Si-NMR (500 MHz, $CDCl_3$, ppm) δ: -65.8. FTIR (cm^{-1}): 3345 (-NH-CO-NH-), 3056 (C-H, sp2), 2922 (C-H, sp^3), 1436 (C-N), 1088 (Si-O-Si), 1000 (Si-O-Si), 733 (Si-C). MALDI-TOF (m/z): $[M-4H]^+$ = 4267.39 observed; $[M]^+$ = 4271.46 calcd.

Synthesis of Octaaminopropyl-POSS-Porphyrin (POSSP-4)

Synthesis of Octaaminopropyl-POSS (OA-POSS) (1,3,5,7,9,11,13,15-octa-(3-amino)-propyl pentacyclo[9.5.1.1(3,9).1(5,15).1(7,13)]octasiloxane) (5)

The synthesis of compound **5** was carried out as previously reported with slight modifications [4]. To a 50-mL round bottom flask containing methanol (24 mL), APTES (3 mL, 12.82 mmol) was added and the mixture stirred at room temperature for 5 min. After the time has elapsed, HCl (4.05 mL, 37% m/v) was added dropwise in the solution (addition time: 5 min). The reaction stirred at room temperature for 7 days. The product was purified via centrifugation, washing twice with methanol. Compound **8** was stored in the freezer at -4 $°C$[4] (Yield = 45.5% wt).

^{1}H NMR (300 MHz, DMSO-D_6, ppm) δ: 8.2 (s, 3H; $-NH_3^+$), 2.79 (t, 2H; $-CH_2NH_3^+$), 1.74 (m, 2H; $-CH_2CH_2CH_2NH_3^+$), 0.74 (t, 2H; Si-CH_2); ^{13}C NMR (300 MHz, DMSO, ppm) δ: 40.7 ($-CH_2NH_3^+$), 21.3 ($-CH_2CH_2NH_3^+$), 9.2 (Si-CH_2); ^{29}Si NMR (500 MHz, DMSO-d6 with 0.1% TMS, ppm) δ: -69.2. FTIR (cm-

^{1}): 3134 (N-H), 2944 and 2876 (C-H), 1489 (C-N), 1098 (Si-O-Si$_{i}$). ESI-MS (m/z): $[M-2H]^{2+}$ = 441.19 observed; $[M]^{2+}$ = 440.14 calcd.

Synthesis of 5-[4-carboxyphenyl]-10,15,20-triphenylporphyrin (Porphyrin-COOH) (6)

The synthesis of compound **6** was carried out as previously reported with slight modifications [5]. In a 1000-mL round bottom flask, CH_2Cl_2 (700 mL) was degassed using nitrogen gas for 30 min. Pyrrole was purified by using a Pasteur pipet with silica gel. This process yields a transparent solution as indication that the polypyrrole has been removed. Purified pyrrole (3.5 mL, 50 mmol), benzaldehyde (3.8 mL, 37.5 mmol) and 4-formylbenzoic acid (1.8768 g, 12.5 mmol) were sequentially added to the flask. Then, boron trifluoride etherate ($BF_3{\cdot}Et_2O$, (0.2 mL, 1.6 mmol) was immediately added to the reaction flask, which was covered with aluminum foil. The solution was stirred at room temperature for 24 h under nitrogen atmosphere. After the completion of the first step, p-chloranil (9.229 g, 37.5 mmol) was added to the flask and the reaction stirred for 19 h at room temperature, in dark conditions. After the reaction completed, the tetrachloro-hydroquinone byproduct was filtered through gravimetric filtration. The solvent was removed with rotary evaporation. The remaining solid was purified using a column chromatography on silica gel (DCM:Ethanol; 97:3). After purification, compound **6** was dried and obtained as a dark purple powder (Yield= 25.3% wt).

^{1}H NMR (500 MHZ, $CDCl_3$, ppm) δ = 8.89 (d, 2H), 8.77 (d, 2H), 8.85 (s, 4H), 8.45 (d, 2H), 8.32 (d, 2H), 8.22 (m, 6H), 7.76 (m, 9H), −2.77 (s, 2H). FTIR (cm^{-1}): 3700-2400 (COOH), 1692.4 (C=O), 1600 (C=C). MALDI-TOF (m/z): $[M-1]^+$ = 659.02 observed; $[M]^+$ = 658.24 calcd.

Synthesis of 5-[4-(succinimidyloxycarbonyl)phenyl]-10,15,20-triphenylporphyrin (Porphyrin-NHS) (7)

The synthesis of compound **7** was carried out as previously reported with slight modifications [5]. In a 25-mL round bottom flask, compound **10** (40.0 mg, 0.06 mmol) was reacted with NHS (8.3 mg, 0.07 mmol) in CH_2Cl_2 (10 mL) in the presence of DCC (18.6 mg, 0.09 mmol) and DMAP (7.3 mg, 0.06 mmol). The reaction was stirred at room temperature for 72 h, under nitrogen atmosphere. Once the reaction was completed, the dicyclohexylurea byproduct was filtrated by gravimetric filtration and the CH_2Cl_2 was dried using a rotary evaporation. Finally, the crude product was purified using column chromatography on silica gel (DCM:Methanol; 9:1). A dark brown solid was obtained after evaporation of the solvent (Yield= 22.6% wt).

^{1}H NMR (500 MHZ, $CDCl_3$, ppm) δ = 8.77-8.90 (m, 8H), 8.52 (d, 2H), 8.37 (d, 2H), 8.24 (m, 6H), 7.76 (m, 9H), 2.94 (brs, 4H), −2.77 (s, 2H). FTIR (cm^{-1}): 3319 (N-H), 2916 and 2848 (C-H), 1738 (C=O), 1603 (C=C). MALDI-TOF (m/z): $[M]^+$ = 755.29 observed; $[M]^+$ = 755.23 calcd.

Synthesis of 5-(4-[3-(3-1,3,5,7,9,11,13,15-octa-(3-amino)-propyl pentacyclo[9.5.1.1(3,9).1(5,15).1(7,13)]octasiloxane) propyl) amido phenyl)-10,15,20-(triphenyl)porphyrin) (POSSP-4)

In a 20-mL round bottom flask compound **5** (9.2 mg, 0.0078 mmol) was mixed in DMSO (10 mL) with DIPEA (7.1 mg, 9.6 µL, 0.0546 mmol). The solution was stirred at room temperature for 30 min. Next, compound **7** (11.8 mg, 0.0156 mmol) was added to the reaction mixture and the reaction stirred at room temperature for 24 h. Once the reaction was completed, the resulting mixture was filtrated with THF and stored in DMSO at -4 $°C$. The final product was purified by using column chromatography on silica gel (DCM:MeOH; 15:1). After purification, compound **11** was dried and obtained as a dark red powder. (Yield = 53.2% wt)

^{1}H NMR (500 MHZ, DMSO-D_6, ppm) δ = 8.77 (m, 8H), 8.22 (m, 10H), 7.76 (m, 9H), 3.00 (t, 2H), 2.75 (t, 14H), 1.65 (m,16H), 0.65 (t, 16H), −3.0 (s, 2H); ^{29}Si NMR (500 MHz, DMSO-D_6, with 0.1% TMS, ppm) δ = -61.2, -69.6, -70.9. FTIR (cm^{-1}): 3385 (N-H), 2992.8 and 2914 (C-H), 1658.6 (C=O), 1425 (C-N), 1279 (Si-C), 1113 (Si-O-Si), 899 (Si-O-Si), 822 and 758 (Si-C).

Synthesis of octa-propylammonium-POSS-Porphyrin (POSSP-5)

Synthesis of octa-propyl trimethylammonium-POSS (Paolo) (IUPAC name: 1,3,5,7,9,11,13,15-octa-(N-methyl-propylammonium) pentacyclo[9.5.1.1(3,9).1(5,15).1(7,13)]octasiloxane (8)

The reaction was performed using a previous protocol with slight modifications.[6] In a 50-mL round bottom flask, potassium carbonate (1.4 g, 10 mmol) was dispersed in acetonitrile (25 mL), and compound **5** (500 mg, 0.43 mmol) was added. The solution was sonicated for 30 min at 40 $°C$. After that, iodomethane (0.55 mL, 13.5 mmol) was added dropwise to the mixture. The temperature was increased 82 °C to using an oil bath. The solution was stirred for 48 h and kept under reflux. The final product was obtained after extraction from methanol and washing with acetone (three times). The solvent was removed with a rotary evaporator to afford a white solid (Yield = 63.4 % wt).

^{1}H NMR (500 MHz, D_2O, ppm) δ: 3.19 (m, 2H; -C*H*$_2$N$^+$(CH$_3$)$_3$), 2.97 (s, 9H; -N$^+$(C*H*$_3$)$_3$), 1.74 (m, 2H; -CH2C*H*2CH2N$^+$(CH$_3$)$_3$), 0.45 (t, 2H; Si-C*H*$_2$); ^{13}C NMR (300 MHz, DMSO-d$_6$, ppm) δ: 68.89 (s, -*C*H$_2$N$^+$(CH$_3$)$_3$), 52.97 (s, -CH$_2$N$^+$(*C*H$_3$)$_3$), 16.71 (s, -*C*H$_2$CH$_2$N$^+$(CH$_3$)$_3$), 8.61 (s, Si-CH$_2$); ^{29}Si NMR (500 MHz, D_2O, ppm) δ: -67.2 (s, T^3). FTIR (cm^{-1}): 3532 (N-H), 2926 (C-H), 1445 (C-N), 1379 (Si-C), 1032 (Si-O-Si), 919 (Si-O-Si).

Synthesis of 5-(4-[3-(1,3,5,7,9,11,13,15-octa-(N-methyl-propylammonium) pentacyclo[9.5.1.1(3,9).1(5,15).1(7,13)]octasiloxane) propyl) amido phenyl)-10,15,20-(triphenyl)porphyrin) (POSSP-5)

The reaction was carried out following the previously described procedure with slight modifications [6]. In a 10-mL round bottom flask, potassium carbonate (0.1 g, 0.724 mmol) was dispersed in acetonitrile (5 mL), and **POSSP-4** (30 mM, 6 mL mg, 0.18 mmol) was added. The solution was sonicated for 30 min at 40 $°C$. After that, iodomethane (1.1 mL, 27 mmol) was added dropwise to the mixture. The temperature was increased 82 °C to using an oil bath. The solution was stirred for 48 h and was kept under reflux. The final product was obtained after extraction from methanol and washing with acetone (three times). The solvent was removed with a rotary evaporator to afford a white solid (Yield = 81.4 % wt).

^{1}H NMR (500 MHZ, DMSO-D$_6$, ppm) δ = δ = 8.67 (m, 8H), 8.48 (d, 2H), 8.32 (d, 2H), 8.25 (m, 6H), 7.65 (m, 9H), 3.35 (m, 16H), 2.94 (s, 63H), 1.93 (m, 16H), 0.39 (t, 16H), −3.0 (s, 2H); ^{29}Si NMR (500 MHz, DMSO-D$_6$, with 0.1% TMS, ppm) δ = -61.2, -69.6, -70.9. FTIR (cm^{-1}): 3323 (N-H); 2967 and 2937 (C-H); 1660 (C=O); 1428 (C-N); 1017 (Si-O-Si); 931 (Si-O-Si), 822 and 758 (Si-C).

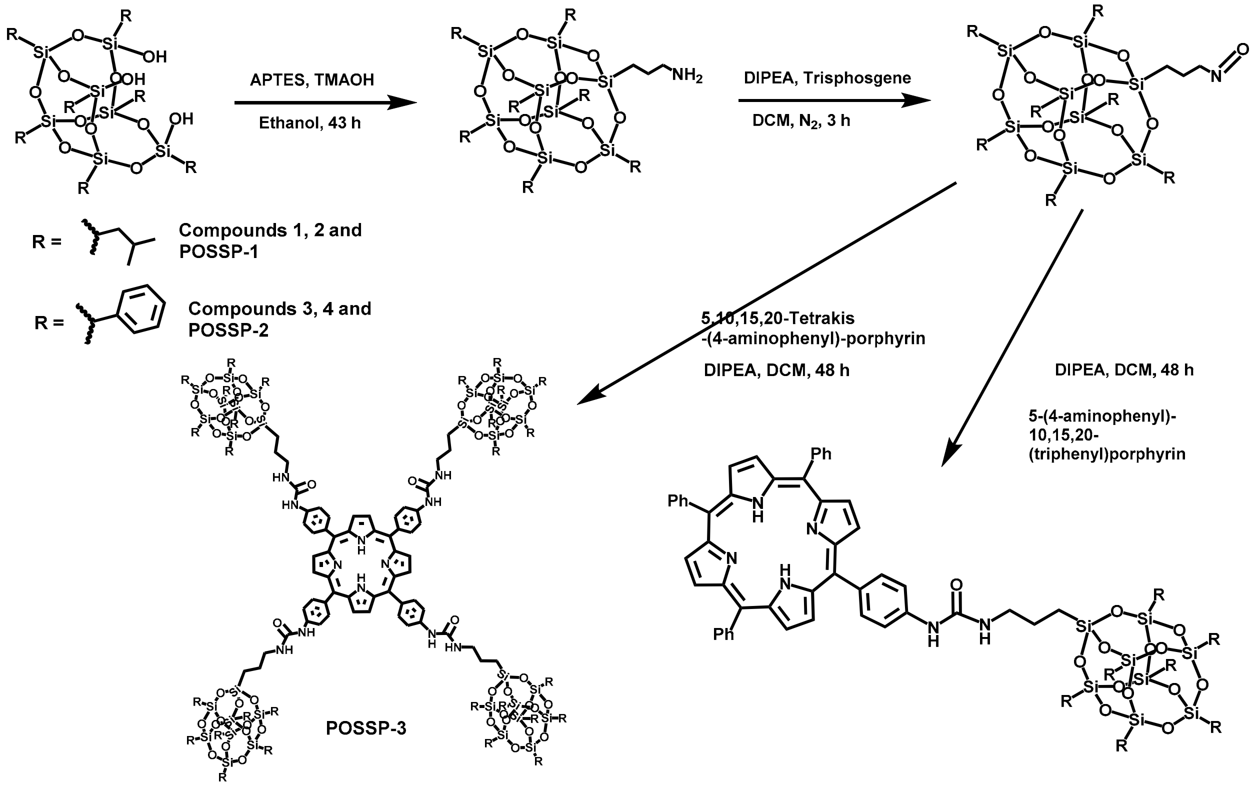

Scheme S1. Synthetic scheme for the synthesis of **POSSPs 1**-**3**. The synthesis of **POSSPs 1** and **2** follow a similar approach. Heptaisobutyl- or heptapheyl-trisilanol POSS was first functionalized with aminopropyl trimethoxysilane under basic conditions, followed by transforming the amine to a cyanate group using triphosgene. Finally, 5-(4-aminophenyl)-10,15,20(triphenyl)porphyrin reacted with either the isobutyl (compound **2**) or the phenyl (compound **4**) version of POSS cyanate derivative to afford **POSSP 1** or **2**, respectively. The synthesis of **POSSP 3** was carried out by reacting compound **2** with 5,10,15,20-tetrakis(4-aminophenyl)porphyrin.

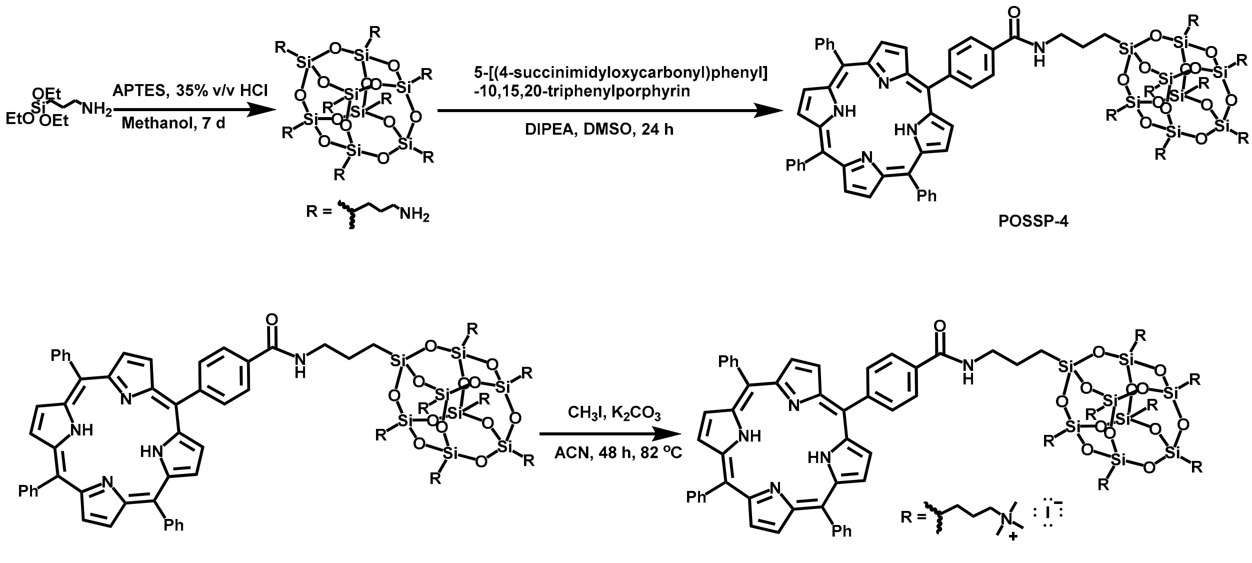

Scheme S2. Synthetic scheme for the synthesis of **POSSPs 4** and **5**. First, octaaminopropyl-POSS (compound **5**) was synthesized using aminopropyltriethoxy silane (APTES). This compound was reacted with compound **7** to afford **POSSP 4**. **POSSP 5** was obtained after the methylation of **POSSP 4**.

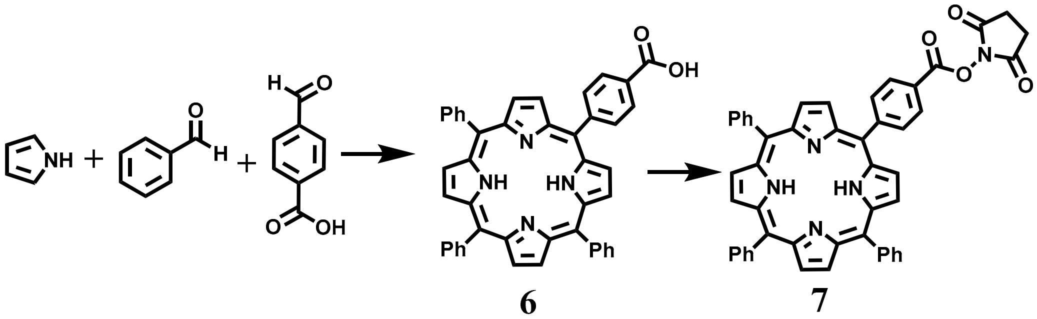

Scheme S3. Synthetic scheme for the synthesis of compounds **6** and **7**.

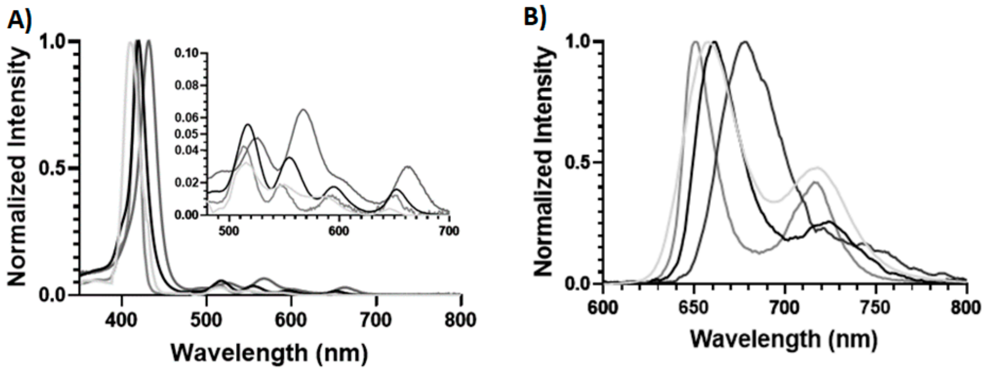

Figure 1. (A) Normalized absorption; and (B) emission spectra for 6.6 μM solutions of parent porphyrins ATPP (black), TAPP (dark gray) and TPP (gray) in THF and 100 μM solutions compound **6** (light gray) in DMSO. The four Q absorption bands are shown in the inset.

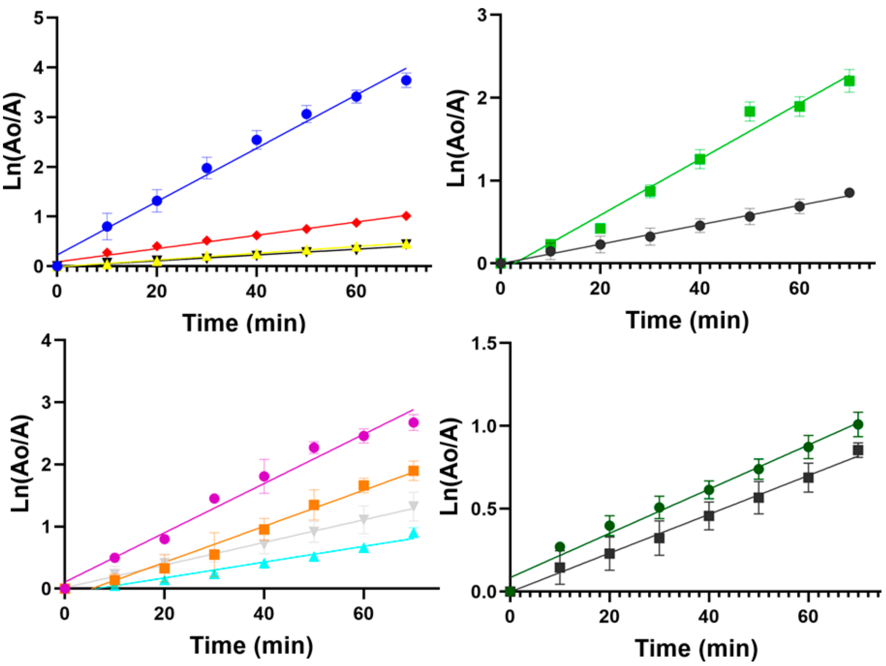

Figure 2. Time-dependent decomposition of DMA plots. **POSSPs 1** (blue), **2** (red), **3** (green), **4** (purple) and **5** (orange); ATPP (black);, TAPP (dark gray); compound **6** (light gray); tetra-hydroxyphenyl porphyrin (dark green); compound **2** + ATPP (yellow); and compound **5** + **6** (aqua).

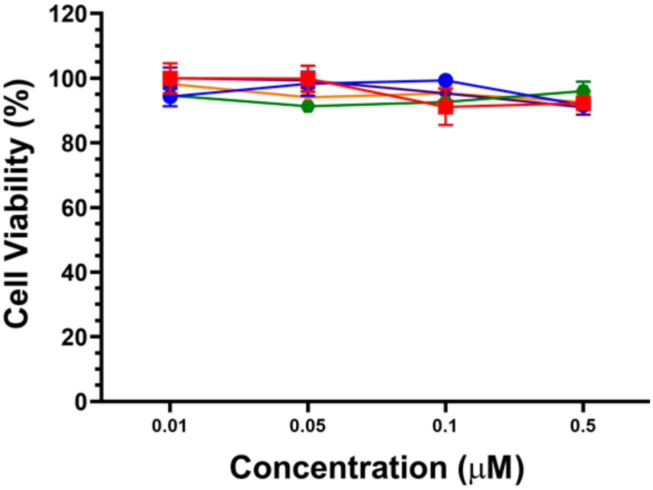

Figure S3. Cytotoxicity of **POSSPs 1** (blue), **2** (red), **3** (green), **4** (purple) and **5** (orange) in MDA-MB-231 cells.

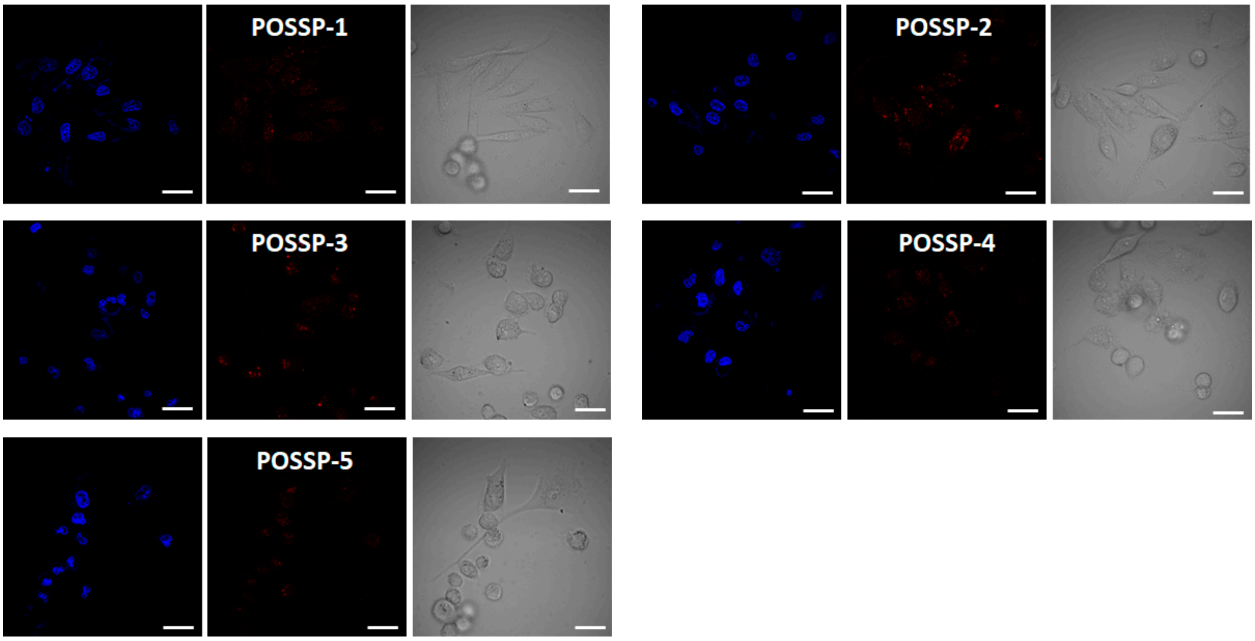

Figure 4. Confocal micrographs of MDA-MB-231 cells inoculated with **POSSPs**. The cell nuclei are observed in the blue channel after staining with Hoechst 33342. The fluorescence in the red channel shows the presence of **POSSPs**. DIC channel depicts the morphology of MDA-MD-231 cells. Scale bar, 30 μm.

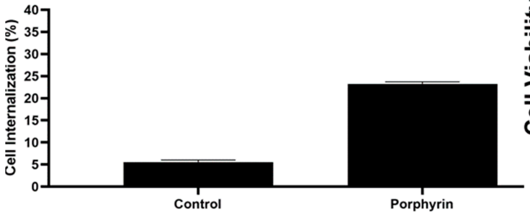

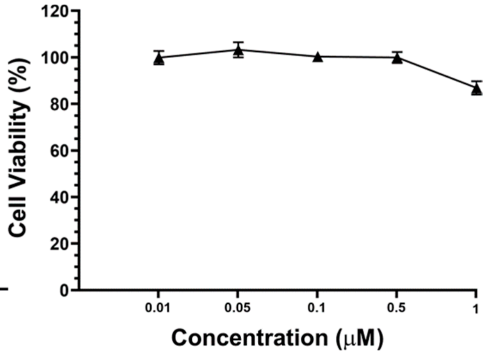

Figure 5. Flow cytometry and cytotoxicity of porphyrin control ATPP.

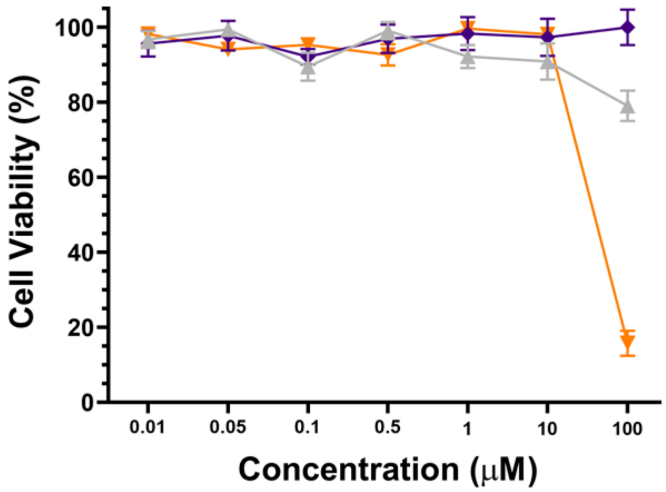

Figure 6. Cytotoxicity of **POSSPs 4** (purple) and **5** (orange) and compound **6** (light gray) in MDA-MB-231 cells in the absence of light.

Table S1. Photophysical and photochemical properties of parent porphyrins.

Sample	λ_{Soret} (nm) [ε x 10^3 (M^{-1} cm^{-1})] (n=3)	$\lambda_{Emission}$ (nm)	Φ_Δ (n=3)	Φ_F (n=3)
**TPP**	416, 434 ± 30	651, 717	0.62	0.12
**ATPP**	419, 275 ± 26	661, 745	0.45 ± 0.02	0.21 ± 0.02
**TAPP**	431, 154 ± 32	678, 787	0.59 ± 0.01	0.16 ± 0.01
**Compound 6**	416, 0.115 ±0.003	650, 710	0.64 ± 0.01	0.13 ± 0.01
**Compound 2 + ATPP**	N/A	N/A	0.53 ±0.01	N/A
**Compound 5 + Compound 6**	N/A	N/A	0.60 ± 0.01	N/A

Table 2. Partition coefficients.

Compound	Log P_{OW}
**ATPP**	3.18 ± 0.08
**TAPP**	1.17 ± 0.06
**Compound 6**	1.20 ± 0.02
**POSSP-1**	0.62 ± 0.08
**POSSP-2**	0.80 ± 0.04
**POSSP-3**	-1.10 ± 0.05
**POSSP-4**	-0.41 ± 0.01
**POSSP-5**	-0.77 ± 0.05

References

1. Zhou, J.L.; Zhao, Y.C.; Yu, K.C.; Zhou, X.P.; Xie, X.L. Synthesis, thermal stability and photoresponsive behaviors of azobenzene-tethered polyhedral oligomeric silsesquioxanes. *New Journal of Chemistry* **2011**, *35*, 2781-2792, doi:10.1039/c1nj20577c.
2. York, M.; Evans, R.A. The use of polyhedral oligomeric silsesquioxane (POSS) as a soluble support for organic synthesis: A case study with a POSS-bound isocyanate scavenger reagent. *Tetrahedron Letters* **2010**, *51*, 4677-4680, doi:10.1016/j.tetlet.2010.07.015.
3. Wheeler, P.A.; Fu, B.X.; Lichtenhan, J.D.; Jia, W.T.; Mathias, L.J. Incorporation of metallic POSS, POSS copolymers, and new functionalized POSS compounds into commercial dental resins. *Journal of Applied Polymer Science* **2006**, *102*, 2856-2862, doi:10.1002/app.24645.
4. Janeta, M.; John, L.; Ejfler, J.; Szafert, S. High-Yield Synthesis of Amido-Functionalized Polyoctahedral Oligomeric Silsesquioxanes by Using Acyl Chlorides. *Chemistry-a European Journal* **2014**, *20*, 15966-15974, doi:10.1002/chem.201404153.
5. Matsumoto, J.; Matsumoto, T.; Senda, Y.; Shiragami, T.; Yasuda, M. Preparation and characterization of porphyrin chromophores immobilized on micro-silica gel beads. *Journal of Photochemistry and Photobiology A: Chemistry* **2008**, *197*, 101-109, doi:https://doi.org/10.1016/j.jphotochem.2007.12.010.
6. Manickam, S.; Cardiano, P.; Mineo, P.G.; Lo Schiavo, S. Star-Shaped Quaternary Alkylammonium Polyhedral Oligomeric Silsesquioxane Ionic Liquids. *European Journal of Inorganic Chemistry* **2014**, 10.1002/ejic.201402005, 2704-2710, doi:10.1002/ejic.201402005.
